# Global and Regional Prevalence of Domestic Violence During the COVID-19 Pandemic and Its Determinants: Protocol for a Systematic Review and Meta-Analysis

**DOI:** 10.2196/60963

**Published:** 2024-12-09

**Authors:** Razieh Bidhendi-Yarandi, Akbar Biglarian, Farhad Nosrati Nejad, Payam Roshanfekr, Samira Behboudi-Gandevani

**Affiliations:** 1 Social Determinants of Health Research Center University of Social Welfare and Rehabilitation Sciences Tehran Iran; 2 Psychosis Research Center University of Social Welfare and Rehabilitation Sciences Tehran Iran; 3 School of Social Health Department of Biostatistics and Epidemiology University of Social Welfare and Rehabilitation Sciences Tehran Iran; 4 Department of Social Welfare University of Social Welfare and Rehabilitation Sciences Tehran Iran; 5 Social Welfare Management Research Center University of Social Welfare and Rehabilitation Sciences Tehran Iran; 6 Faculty of Nursing and Health Sciences Nord University Bodø Norway

**Keywords:** COVID-19, domestic violence, systematic review and meta-analysis, lockdowns, pandemic effects, intimate partner violence, elder abuse, child abuse, vulnerable populations

## Abstract

**Background:**

Domestic violence is one of the most significant global public health priorities. This social problem could be accelerated by global catastrophes such as the COVID-19 pandemic. The structural changes due to the imposition of health measures, combined with personal and social problems, may worsen the situation.

**Objective:**

This study aims to investigate the global and regional prevalence of domestic violence during the COVID-19 pandemic and its determinants.

**Methods:**

We will perform a comprehensive review of the literature in PubMed, PsycINFO, Embase, Cochrane COVID-19 Register, and Applied Social Sciences Index and Abstracts, up to July 2024. This review will adhere to the PRISMA (Preferred Reporting Items for Systematic Reviews and Meta-Analyses) reporting guidelines. Observational studies will be considered eligible if they have a population-based design, report the number of cases or prevalence of domestic violence during the COVID-19 pandemic, and report potential determinants. Studies in languages other than English, those with unclear data, case reports, conference proceedings, reviews, and letters will be excluded. To assess the methodological quality, a standardized critical appraisal checklist for studies reporting prevalence data will be used. A robust Bayesian approach will be applied using the STATA software package (version 14; STATA Inc) and JASP 0.19.1 (GNU Affero General Public License [GNU AGPL]) software.

**Results:**

The search and screening for the systematic literature review are anticipated to be finished in October 2024. Data extraction, quality appraisal, and subsequent data synthesis will begin in November 2024. The review is expected to be completed by April 2025, and the study results will be published in 2025.

**Conclusions:**

This systematic review and meta-analysis will address significant gaps in understanding the pandemic’s impact on domestic violence, providing a comprehensive assessment of its prevalence and contributing factors. Despite some limitations, the study incorporates diverse data sources and vulnerable groups to offer a detailed and accurate picture. The findings will inform targeted interventions and policy responses to mitigate the impact of future global crises on domestic violence rates.

**Trial Registration:**

PROSPERO CRD42022351634; https://tinyurl.com/yth37jkx

**International Registered Report Identifier (IRRID):**

PRR1-10.2196/60963

## Introduction

Domestic violence (DV) is one of the most important global public health priorities. It can include physical, sexual, psychological, and financial dimensions, as well as controlling or coercive actions and behaviors by one or more members against others of a household [1]. Usually, women are survivors of DV by their intimate partners [2]. It is showed that 1 in 3 women will be a survivor of physical or sexual violence during her life [3]. Despite efforts to decrease it, DV still occurs worldwide and may be amplified by factors such as natural disasters and socioeconomic crises [4-7].

The COVID-19 pandemic, as a global health crisis, brought the greatest challenges in all aspects of human life. It has caused structural changes in the category of human communication and has resulted in many adverse psychosocial effects [8-10]. Evidence shows that the levels of DV increased globally as the COVID-19 pandemic escalated. According to the World Health Organization (WHO) Eastern Mediterranean Region (EMRO), the prevalence of violence against women in the region is the second highest in the world at 37% [11]. The National Commission on COVID-19 and Criminal Justice also reported that DV incidents in the United States increased by 8.1% after the enforcement of the lockdown [12].

Factors such as quarantine, staying at home, remote work, and many other imposed conditions, in addition to personal and social crises, put pressure on people beyond the threshold of tolerance, which may exacerbate DV [13-19]. DV during the COVID-19 pandemic remains as an important health concern and a serious human rights violation, and addressing it is still a global issue. In addition, identifying critical gaps of knowledge to develop response strategies, principles, and recommendations for controlling DV during long- and short-term catastrophic events like COVID-19 is essential for public health emergencies [20-23]. There are some review studies that addressed this issue [24-29]. However, our study would be the first to provide pooled estimates of global and regional prevalence of DV during COVID-19 and measure the effect of determinants using strong statistical methodology.

The purpose of this systematic review and meta-analysis is to investigate the global and regional prevalence of DV during the COVID-19 pandemic and its associated determinants.

## Methods

### Overview

This study adheres to the PRISMA (Preferred Reporting Items for Systematic Reviews and Meta-Analyses) reporting guidelines. The study protocol was followed PRISMA-Protocol (PRISMA-P; Multimedia Appendix 1). The protocol of this study was registered in PROSPERO (CRD42022351634)

The review question was framed based on the PICO (Population, Intervention, Comparison, and Outcomes) statement as follows:

- P: Populations affected by COVID-19, as defined by the studies, will be included. There will be no limits in terms of gender or ethnicity.
- I: This review will consider studies that report on the prevalence of DV during the COVID-19 pandemic.
- C: This study does not include a comparison.
- O: The primary outcome of study is pooled prevalence of DV during COVID-19. This includes physical violence, sexual, psychological, controlling behavior violence, and economic violence.

As secondary outcomes, all factors and determinant associated with DV will be extracted. The determinants can be but are not limited to age, gender, educational status, decision-making power, social support, wealth index, and unemployment. In cases of missing data or articles with no available full text, we will contact the corresponding author 3 times. Articles will be excluded if no response is received.

### Eligibility Criteria

Observational studies will be considered eligible if they have a population-based design, report the number of cases or prevalence of DV during the COVID-19 pandemic, and report potential determinants. Studies in languages other than English, those with unclear data, case reports, conference proceedings, reviews, and letters will be excluded.

### Search Strategy

We will conduct a comprehensive literature review using databases such as PubMed (including Medline), Embase, PsycINFO, Google Scholar, Cochrane COVID-19 register, and Applied Social Sciences Index and Abstracts, up to July 2024. In addition, we will manually search the reference lists of selected studies and other relevant reviews to maximize the identification of eligible studies and consider results from gray literature.

### Search String

To identify relevant studies for our systematic review and meta-analysis, we will develop and execute a comprehensive search strategy across multiple electronic databases. Our search will incorporate Medical Subject Headings (MeSH), keywords, and specific search terms as outlined in Table 1. The search strategy will be tailored to each database to ensure optimal retrieval of relevant articles. The search strategy will be adapted to align with the database’s indexing and search capabilities. Specifically, in PubMed we will use MeSH terms in combination with keywords. The search string will incorporate MeSH terms and keywords to capture the breadth of literature related to DV during the COVID-19 pandemic (“Domestic Violence”[MeSH] OR “Spouse Abuse”[MeSH] OR “Elder Abuse”[MeSH] OR “Child Abuse, Sexual”[MeSH] OR “Intimate Partner Violence”[MeSH] OR “Physical Abuse”[MeSH]) AND (“child*” OR “Infant” OR “adolescen*” OR “young people” OR “adults” OR “Women” OR “Frail Elderly” OR “Aged”) AND (“Coronavirus” OR “COVID-19” OR “SARS-CoV-2” OR “2019 novel coronavirus”) AND (“prevalence” OR “incidence” OR “rate” OR “diagnosis” OR “disease burden” OR “epidemiology” OR “frequency” OR “determinants” OR “predictors” OR “correlates” OR “risk factors” OR “protective factors”). For other databases, keywords and subject headings relevant to each database’s search functionality will be used.

**Table 1:** Keywords and search strategy.

Search query	Search topic	Search keywords (titles, abstracts, general keywords, and subject headings)
1	Condition or outcome of interest	“Domestic Violence” OR “Spouse Abuse” OR “Elder Abuse” OR “Child Abuse, Sexual” OR “Child Abuse” OR “Intimate Partner Violence” OR “intimate terrorism” OR “coercive controlling violence” OR “Physical Abuse” OR “Homicide” OR “verbal abuse” OR “sexual abuse” OR “Sexual assault” OR “physical assault” OR “verbal assault” OR “Family violence” OR “Intimate Partner Aggression” OR “intrepersonal violence” OR “partner violence” OR “Domestic harassment”
2	Population	No limitation
3	Exposure or context	“Coronavirus” OR “COVID-19” OR “COVID19” OR “SARS-CoV-2” OR “2019 novel coronavirus” OR “2019-nCoV” OR pandemia OR “global pandemic” OR lockdown
4	Phenomenon	No limitation
Final search query	Intersection of 4 topics	1 AND 3

### Study Selection and Data Extraction

The titles, abstracts, and full texts of selected studies will be screened independently by 2 authors based on the eligibility criteria. The following data will be extracted from eligible studies: the first author’s name, publication year, study design, sample size, characteristics of population, determinants, and outcome measurements. The accuracy of data before the meta-analysis will be assessed by double-checking the data extraction process to ensure no bias in the data extraction and data entry. Any discrepancies will be resolved through discussion with the senior review author.

### Assessment of Methodological Quality

We will use the Joanna Briggs Institute (JBI) Critical Appraisal Checklist [30] for Prevalence Studies, which includes criteria for evaluating aspects such as study design, sample selection, measurement methods, and statistical analysis. Two independent reviewers will conduct the methodological quality assessment of each included study. Each reviewer will assess the studies separately to ensure a thorough evaluation. Any disagreements between the reviewers regarding the appraisal of study quality will be resolved through discussion. If consensus cannot be reached, a third reviewer, who is not involved in the initial assessment, will be consulted to adjudicate and provide a final decision. The results of the quality assessments will be documented and used to inform the interpretation of the review’s findings. Studies will be classified based on their methodological quality to help in the synthesis and analysis of data.

### Statistical Analysis

The STATA software package (version 14; STATA Inc) and JASP 0.19.1 (GNU Affero General Public License [GNU AGPL]) software will be used to conduct statistical analysis. Heterogeneity will be evaluated using the chi-square test and *I*^2^ index. Publication bias will be assessed using funnel plots and statistical tests. In case of significant results, the robust Bayesian approach will be applied. The random effect model will be used for the estimation of the pooled prevalence via the meta-prop method with a pooled estimate after Freeman-Tukey double arcsine transformation to stabilize the variances. Forest plots for each outcome and by any extracted subgroups such as gender, regions, type of DV, educational level, etc, will be illustrated as well. Meta-regression analysis will be run to assess the effect of extracted determinants as the potential sources of heterogeneity. Sensitivity analysis will be applied to find influential studies in case.

### Ethical Considerations

The protocol of this study was registered in PROSPERO (CRD42022351634). This is a systematic review of published papers, which does not require informed consent. In addition, this study was considered by the ethics committee of the University of Social Welfare and Rehabilitation Sciences, Tehran, Iran (ethics code: IR.USWR.REC.1401.140).

## Results

The search and screening for the systematic literature review are anticipated to be finished in October 2024. Data extraction, quality appraisal, and subsequent data synthesis will begin in November 2024. The review is expected to be completed by April 2025, and the study results will be published in 2025.

## Discussion

### Principal Findings

The results of this systematic review and meta-analysis will estimate the prevalence of DV and its determinant in the COVID-19 pandemic and is poised to address critical gaps in the existing literature. It is anticipated that the study will reveal a significant increase in DV rates both globally and regionally, driven by pandemic-related conditions such as lockdowns, economic stress, and restricted access to support services. The analysis is expected to identify critical determinants contributing to this rise, including socioeconomic status, household size, and preexisting conditions of abuse. By illuminating these factors, the study seeks to inform effective interventions and policy responses to mitigate DV in the context of ongoing and future public health crises.

The occurrence of a natural disaster typically leads to an increased risk of social health problems [31]. Disease outbreaks such as COVID-19 pandemic necessitate emergency prevention and control measures in the community; however, they also result in psychosocial side effects [29,32-34].

Generally, there are several potential factors associated with DV, including individual traits such as age; ethnicity; previous history of exposure or experience of violence; psychological disorders such as depression, anxiety, substance abuse, and suicidality; as well as sociocultural factors that foster a culture of violence against women and social norms supporting male authority and control over women [24,35,36].

Evidence showed that COVID-19 preventive interventions such as physical distancing, lockdown, and social isolation were also associated with psychiatric problems, including anxiety, depression, suicidal thoughts, and DV [25,26,37-39]. A systematic review study that included 18 studies showed an increase in reported incidents of DV due to COVID-19–related restrictions, such as stay-at-home and lockdown orders [12]. Another integrative review that included 38 studies also indicated some factors increasing women’s vulnerabilities to violence, such as social distancing and lockdown [40]. It seems that COVID-19 preventive and controlling policies as a mediator factor can exacerbate potential related factors, leading to an escalation of violence and abusive behaviors. Therefore, results of studying the dynamics of this violent behavior, its associated factors, and COVID-19 measures can be extrapolated to global disaster situations [41].

In addition, Kifle et al [42], in a systematic review of 14 observational studies, estimated the prevalence of intimate partner violence on women during the COVID-19 pandemic. They reported that nearly 1 in 3 women experienced intimate partner violence during the COVID-19 pandemic. Subgroup analysis based on region showed that the highest prevalence of intimate partner violence was in resource-limited regions (33%). All forms of intimate partner violence (physical, sexual, emotional, and economic) were prevalent.

However, some limitation in those studies, such as narrow inclusion criteria, often focusing exclusively on women, thereby neglecting other vulnerable groups such as older individuals. Furthermore, important databases such as Embase were not included in some available studies, potentially limiting the scope and robustness of their findings. In addition, most of them did not report the determinants and risk factors for DV.

Crisis management organizations, health care providers, and policy makers should be aware of the potential increase in rate of DV during state of emergency. They should provide suitable health care facilities such as local referral pathways of services, support and counseling services, protection services, hotlines, and shelters. Humanitarian and nongovernment organizations could also step up to provide necessary services to support DV survivors during crises.

The strength of our study lies, first, in the breadth of evidence it produces. Bayesian meta-analysis will be applied to address potential publication bias, a critical mythological concern in prevalence meta-analysis [43,44]. Second, we will identify significant determinants through a meta-regression approach. Additionally, we will ensure high methodological quality by adhering to the AMSTAR (Assessing the Methodological Quality of Systematic Reviews) guideline. However, our study has limitations. DV is widely underreported worldwide, and obstacles to data collection during COVID-19 intensify this issue [45,46]. One limitation regarding the control of violence could be the lack of accurate data and evidence. Therefore, response organizations and authorities need to include support services to facilitate the safe collection of data on reported cases of DV in their crisis plans. Studies included in the meta-analysis may use different definitions and methodologies to assess DV, which could contribute to heterogeneity and affect the reliability of pooled prevalence estimates. DV prevalence and its determinants may vary significantly across different global regions and cultural contexts. Addressing this heterogeneity adequately in the meta-analysis could be challenging but crucial for accurate regional estimates. Access to the full text of the articles may be restricted, which will be resolved by corresponding with authors. In addition, strong heterogeneity among studies may exist, which will be addressed using random effect models and appropriate subgroup analyses.

### Conclusion

This systematic review and meta-analysis will address significant gaps in our understanding of the pandemic’s impact on DV. Although there are some limitations, prior research has highlighted the increase in DV incidents linked to COVID-19 restrictions. This study will provide a comprehensive assessment and analysis, encompassing a broader range of vulnerable groups and incorporating diverse data sources. By doing so, it seeks to offer a more detailed and accurate picture of the prevalence and factors contributing to DV during the pandemic. The findings will be crucial in informing targeted interventions, shaping policy responses, and ultimately mitigating the impact of future global crises on DV rates.

## Appendix

Multimedia Appendix 1 PRISMA-P (Preferred Reporting Items for Systematic Review and Meta-Analysis Protocols) 2015 checklist.

## Abbreviations

AMSTAR: Assessing the Methodological Quality of Systematic Reviews
DV: domestic violence
EMRO: Eastern Mediterranean Region
JBI: Joanna Briggs Institute
MeSH: Medical Subject Headings
PICO: Population, Intervention, Comparison, and Outcomes
PRISMA: Preferred Reporting Items for Systematic Reviews and Meta-Analyses
PRISMA-P: Preferred Reporting Items for Systematic Review and Meta-Analysis Protocols
WHO: World Health Organization
